# Supplementary material for: An Exploratory Study of Large-Scale Brain Networks during Gambling Using SEEG
Source: Brain Sci. 2024 Jul 31;14(8):773. doi: 10.3390/brainsci14080773 (PMC11352602; doi:10.3390/brainsci14080773)
Supplement: Supplementary file 1 [file brainsci-14-00773-s001.zip › brainsci-3083451-supplementary.pdf]

Supplementary Materials

An Exploratory Study of Large-Scale Brain Networks during Gambling Using SEEG

Christopher Taylor [1], Macauley Smith Breault [2,*], Daniel Dorman [1], Patrick Greene [1], Pierre Sacré [3], Aaron Sampson [4], Ernst Niebur [4], Veit Stuphorn [4], Jorge González-Martínez [5] and Sridevi Sarma [1]

[1] Department of Biomedical Engineering, Johns Hopkins University, Baltimore, MD 21218, USA; chris.j.w.taylor94@gmail.com (C.T.); daniel.b.dorman@jhu.edu (D.D.); pagreene@jhu.edu (P.G.); ssarma2@jhu.edu (S.S.)
[2] The Picower Institute for Learning and Memory, Massachusetts Institute of Technology, Cambridge, MA 02139, USA
[3] Department of Electrical Engineering and Computer Science, University of Liège, 4000 Liège, Belgium; p.sacre@uliege.be
[4] Solomon Snyder Department of Neuroscience, Johns Hopkins University, Baltimore, MD 21218, USA; asamps10@jhu.edu (A.S.); niebur@jhu.edu (E.N.); veit@jhu.edu (V.S.)
[5] School of Medicine, University of Pittsburgh, Pittsburgh, PA 15213, USA; gonzalezmartinezja@upmc.edu
* Correspondence: breault@mit.edu

**Network Pair**	**n1**	**n2**	**U**	**Effect Size**	**Power**
Gamma Band					
DMN-DAN	471	673	118980	0.249297	0.968687
DMN-DMN	751	833	244069	0.219707	0.989126
DMN-FN	475	671	99949	0.37282	0.999921
DAN-DAN	339	501	36432	0.570982	1
DAN-FN	339	511	61099	0.294587	0.969249
FN-FN	136	185	9019	0.283068	0.642815
Theta Band					
DMN-DAN	471	673	124347	0.215434	0.910318
DMN-DMN	751	833	237023	0.242233	0.996843
DMN-FN	475	671	138169	0.132989	0.534878
DAN-DAN	339	501	41840	0.507298	1
DAN-FN	339	511	81853	0.054973	0.110257
FN-FN	136	185	10772	0.14372	0.218823

Table S1: Power analysis for connectivity strength comparisons between low and high performers for gamma and theta bands, related to Figure 6. n1 is the number of observations (trials) in the high performer group, n2 is the number of observations (trials) in the low performer group, U is the Mann Whitney U test statistic, the effect size is computed as 1 – (2U)/(n1 n2), and the power column indicates statistical power.

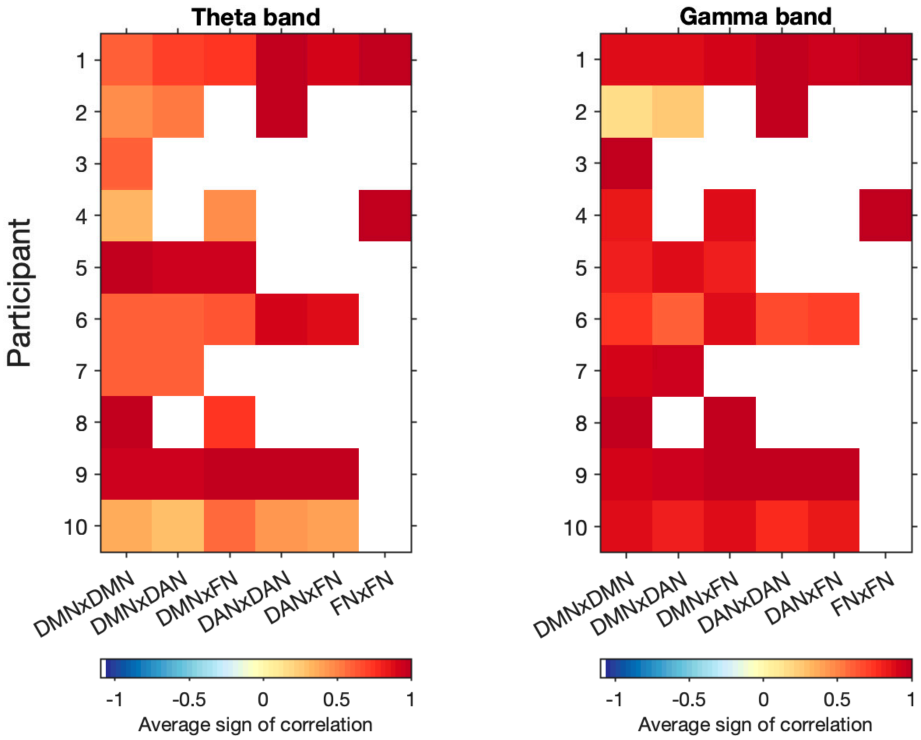

Figure S1: Summary of sign of correlation within and between networks

The average sign of the correlation within and between networks for each participant displayed as a heatmap ranging from -1 (blue) to 1 (red). White means that the participant did not have a pair of networks. We found that the relationships of the connectivity are mostly positive, meaning the power in theta and gamma between each pair of networks would increase together.
